# Controlling parameters and characteristics of electrochemical biosensors for enhanced detection of 8-hydroxy-2′-deoxyguanosine

**DOI:** 10.1038/s41598-019-43680-y

**Published:** 2019-05-15

**Authors:** Aline M. Faria, Elisa B. M. I. Peixoto, Cristiane B. Adamo, Alexandre Flacker, Elson Longo, Talita Mazon

**Affiliations:** 1 Centro de Tecnologia da Informação Renato Archer, CTI, Rod. D. Pedro I, KM 143.6, 13069-901 Campinas, SP Brazil; 2 CDMF, Universidade Federal de São Carlos, P.O. Box 676, São Carlos, SP 13565-905 Brazil

**Keywords:** Sensors and biosensors, Diagnosis, Biosensors

## Abstract

This work discusses the parameters and characteristics required on the development of a scalable and reliable electrochemical sensor board for detecting 8-hydroxy-2′-deoxyguanosine (8-OHdG), an oxidative stress biomarker for diabetic nephropathy, cancer and Parkinson’s disease. We used Printed Circuit Board (PCB) technology to make a precise, low-cost bare sensor board. ZnO nanorods (NRs) and ZnO NRs: reduced graphene oxide (RGO) composites were used as a pathway for antibody immobilization on the working electrode (WE). The parameters and characteristics of the WE were controlled for enhancing the quality of the electrochemical sensor board. Thickness of the gold and the presence of ZnO NRs or their composite on the WE have influence on charge transference process and reproducibility of the sensor board. The amount of the antibody, and its incubation period are crucial to avoid saturation of the sites during immobilization step and reduce the cost of the sensor. Our ZnO NRs-based electrochemical sensor board showed high sensitivity and selectivity to 8-OHdG with detection capacity in the range of 0.001–5.00 ng.mL^−1^. The successful application of our immunosensor to detect 8-OHdG in urine was evidenced.

## Introduction

In recent years, concerns about health spending have become prominent for researchers and governments. Saving an estimate of $1.333 trillion on health may be possible with improvements in disease prevention and treatment^1^. Among diseases of great impact on public health are neurodegenerative diseases, cancer and diabetes. Performing early detection of these diseases is an effective means for successful treatment and reducing mortality and costs^2–4^.

Neurodegenerative diseases, cancer and diabetes lead an overproduction of Reactive Oxygen Species (ROS). The presence of higher concentration of ROS leads hydroxylation of DNA and produces 8-OHdG in the human body^5^. Owing to this, 8-OHdG is recognized as a suitable biomarker of Oxidative Stress^6^, being present in urine^4,7^, saliva^8^, blood^7^, and tissue^9^. Healthy patients show around 0.2 ng.mL-1 8-OHdG in serum. This concentration increases 10-fold as ROS levels increase^10^. Therefore, the development of precise diagnostic tools capable of evaluating the 8-OHdG concentration in real time is necessary^11^.

In this sense, electrochemical biosensors have allured much attention as a promising tool for detection of 8-OHdG. Among the advantages are fast response, excellent cost-effectiveness, ease of handling, and possibility of being portable^12–14^. Considerable amount of work has been devoted to the development of electrochemical biosensors with better sensitivity and selectivity. A three-electrode configuration is used in these biosensors, one working (WE), one reference (RE), and one counter electrode (CE)^15–17^. Normally, these electrodes are fabricated by conventional methods, such as: inkjet-printed^18^, roll to roll^19^, sputtering^20^ and printed circuit board (PCB)^21^. PCB shows the advantages of being relatively cheap and suitable for large scale-manufacturing, since the metallic deposition is made by electroplating. Despite their advantages, few reports describe the importance of controlling the parameters of the manufacturing process, especially of the sensor board, in the improvement of biosensor properties. Most of them just describe how modifying the WE with nanostructures for improving sensitivity and limit of detection. Carbon nanotubes^22–25^, and graphene^24,26,27^ have been commonly reported as good WE for the detection of 8-OHdG. Despite their higher conductivity and specificity, biosensors built with these materials showed sensitivity in the picomolar range. Another problem found in these biosensors is poor selectivity once they suffer to interference from others components present in the blood or urine. One of the ways to overcome these issues is controlling parameters and characteristics of the electrochemical sensing system, including board sensor.

Along these lines, this work aims to control parameters involved in preparing electrochemical biosensors, from the manufacturing of the bare-sensor board. As a result, we prepared biosensors with better sensitivity and selectivity for detecting 8-OHdG. For meeting all these requirements, we used PCB to make the bare-sensor board and zinc oxide nanorods (ZnO NRs) or ZnO (NRs): reduced graphene oxide (RGO) composite as WE. ZnO NRs were used to aid biomolecules immobilization step. They also improve the electron transference rate between the biomolecules and the electrode and, thereby, enhanced the sensitivity of the sensor^28,29^. On the other hand, RGO has been reported as excellent material to provide a uniform distribution of electrochemical active sites improving limit of detection^30^. The characteristics of the working electrode, including thickness of the metallic trail, were here set to find suitable electrical characteristics and reproducibility. Our biosensor could detect 8-OHdG in the human urine without pre-treatment of it, and accuracy (detection in fg.mL^−1^). The possibility of analyzing urine without pre-treatment reduces the costs of accomplishing.

## Results and Discussion

### Bare-sensor board optimization

The characteristics of the working and counter electrodes can have great influence over the quality of the electrochemical sensor board. Particularly, conductivity and reproducibility of the electrodes may affect the sensor response. Gold (Au) electrode is well-known to match excellent conductivity and reproducibility. On the other hand, copper (Cu) is an unstable material; however, it is widely used as metal trails and pads in PCBs.

**Figure 1:**
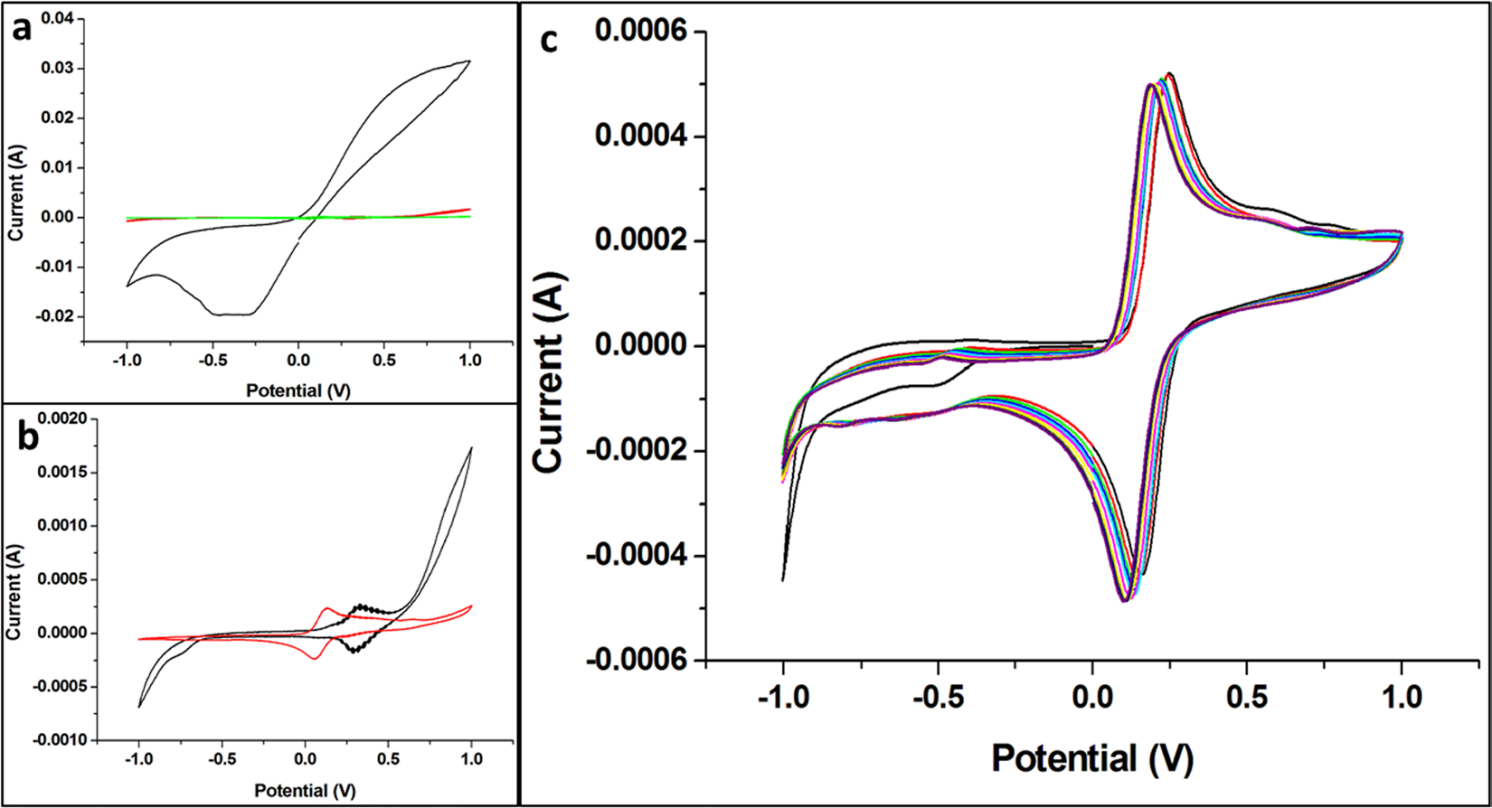
Cyclic voltammograms (CVs) obtained for different bare-sensor boards: (**a**) in black for board made by using Cu-based WE and CE with 1/1OZ thickness, in red and green for Au film-based WE and CE with 0.5 µm and 3.0 µm thickness, respectively. (**b**) Au film-based WE and CE, in black 0.5 µm and in red 3.0 µm thickness, respectively. (**c**) 10 scans for 3.0 µm thickness of Au film-based WE and CE. All CVs were performed in the presence of K_3_[Fe(CN)_6_]/K_4_[Fe(CN)_6_] (10 mM) in NaNO_3_ (0.5 mol.L^−1^), with a scan rate of 100 mV.s^−1^.

In order to evaluate the conductivity and stability of our bare-sensor board, we tested the use of copper or gold films as working and counter electrodes. For bare-board made with copper electrodes, a non-characteristic voltammogram is obtained due to oxidation of the copper (Fig. 1a). Since copper is unstable, using a non-reactive metal, such as gold, may be critical on making stable and reliable electrochemical electrodes. Therefore, we also tested gold deposited by an electrolytic method as WE and CE. Figure 1b shows that metal used, and its thickness influence the stability and response of the bare-sensor board. A 3.0 μm thickness favors obtaining a more stable characteristic cyclic voltammogram compared to 0.5 μm. One of the reasons may be related to the decrease of sheet resistance at higher thickness, increasing the sensor performance. Consequently, we set the thickness of our gold WE and CE as 3 μm.

Another characteristic that must be taken into account in sensor development is the reference electrode. Usually, this electrode is a solid Ag/AgCl, without being integrated with the others. For integrated systems, it is used a layer of silver ink that has been previously immersed into a chloride solution. Herein, the RE was produced by using a silver conductive epoxy which contains chloride ion in its composition. The cyclic voltammograms (CVs) obtained for bar sensor boards using silver conductive epoxy or Ag/AgCl ink as RE are very similar (Fig. S1), suggesting the silver epoxy can be used as RE.

The stability of the bare-sensor board was evaluated by ten successive CVs performed in the presence of K_3_[Fe(CN)_6_]/K_4_[Fe(CN)_6_] (10 mM) in NaNO_3_ (0.5 mol.L^−1^), by using 100 mV.s^−1^ scanning rate and potential range from −1.0 to 1.0 mV (Fig. 1c). The anodic current (Ipa) values were basically constant (coefficient of variation of 0.8%), even after 10 scans. As lower coefficient of variation is, more homogeneous is the data set^31^. The excellent stability of our homemade bare-sensor board can improve properties like sensibility and limit of detection. In addition, the easy of technique and possibility of large-scale production contribute to its potential application as an electrochemical sensor.

### Growth of ZnO NRs or ZnO NRs:RGO composite on Au film-based WE

ZnO NRs, graphene and reduced graphene oxide (RGO) are excellent materials used to anchor biomolecules, like antibodies, in their surface, as well as assisting in the path of the electron^28^. Because of this, we decide to growth ZnO NRs and its composite on WE.

Prior to the growth of ZnO nanostructures on Au film-based WE, a GO/ZnAc-based seeding layer was deposited by spray coating. According to our previous work, this seeding layer provides Zn^2+^ and oxygen functional groups that corroborate for homogeneous growth of ZnO nanostructures^32^.

**Figure 2:**
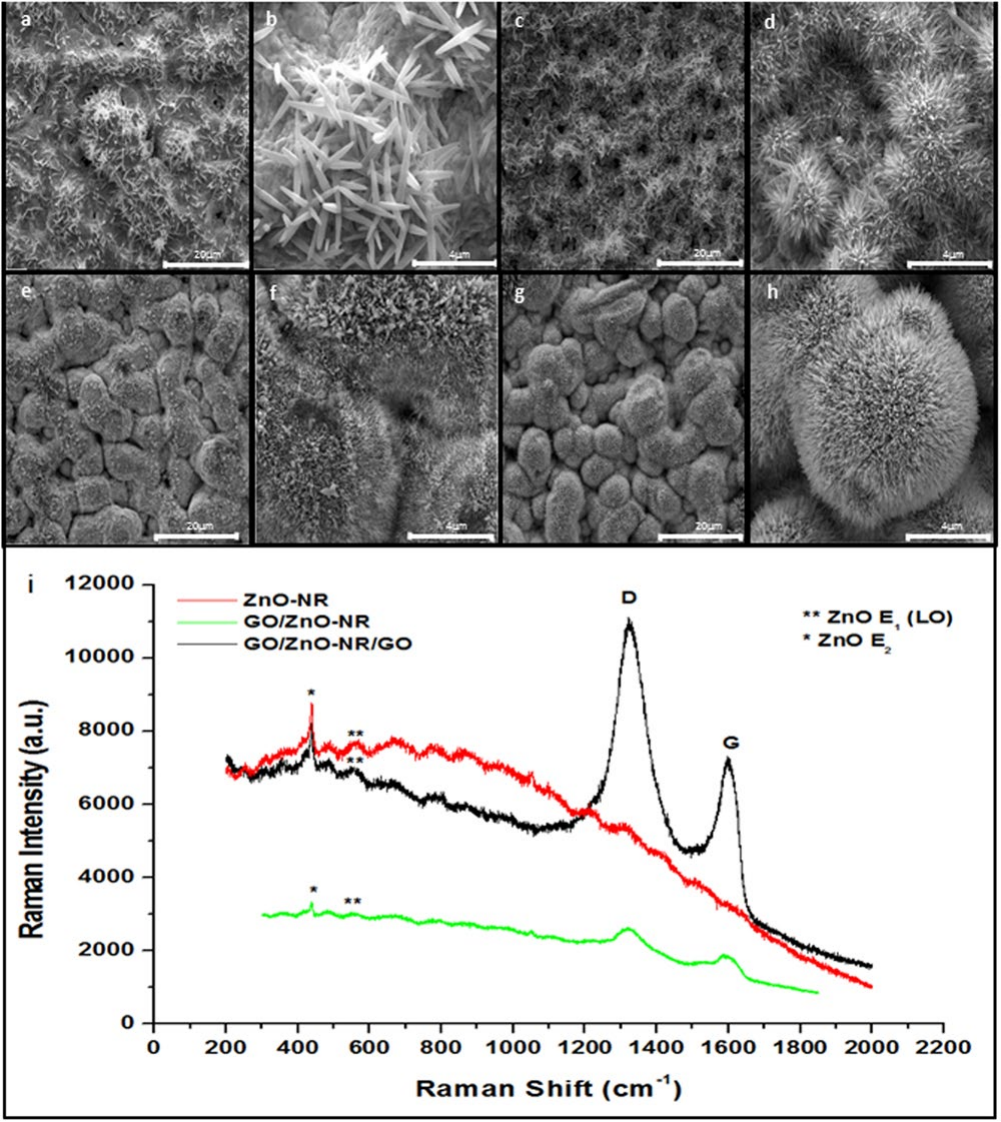
SEM images of the ZnO structures grown on the sensor board: (**a**) and (**b**) without seeding layer (5000x and 25000x, respectively); (**c**) and (**d**) 03GO03ZnAc (5000x and 25000x, respectively); (**e**) and (**f**) 06GO06ZnAc (5000x and 25000x, respectively); (**g**) and (**h**) 12GO12ZnAc (5000x and 25000x, respectively). (**i**) Raman spectra.

For verifying the influence of GO and ZnAc layers on size, density and orientation of the ZnO NRs, we sprayed three, six and twelve layers of each solution. The evaluation of the morphology and density of the nanostructures arrays was followed by SEM (Fig. 2a–h). The absence of seeding layer leads to non-homogeneous growth of microrods (Fig. 2a,b). The increase in the number of sprayed layers helped the growth of ZnO NRs due to the larger number of Zn^2+^ and oxygen functional groups active sites (Fig. 2c–h). Twelve nucleation layers favored growing ZnO NRs with higher density and perpendicularly oriented to the substrate (Fig. 2g,h). Therefore, we set 12GO12ZnAc as the best seeding layer for growing ZnO NRs or ZnO NRs:RGO composite.

All Raman spectra showed the usual modes of ZnO, such as 333 cm^−1^ (E2(high)-E2(low)^33,34^, 437 cm^−1^ (E2(high) characteristics of the wurtzite lattice^35,36^, and 573 cm^−1^ (A1(LO) mode) assigned to the electric field-induced (EFI) Raman scattering^37^ (Fig. 2i). All samples prepared with addition of GO, as seeding layer (named here GO/ZnO NRs) or as seeding layer and composite (named here GO/ZnO NRs/RGO), also showed the D (1300–1400 cm^−1^) and G (1500–1700 cm^−1^) bands referent to GO (Fig. 2i).

**Figure 3:**
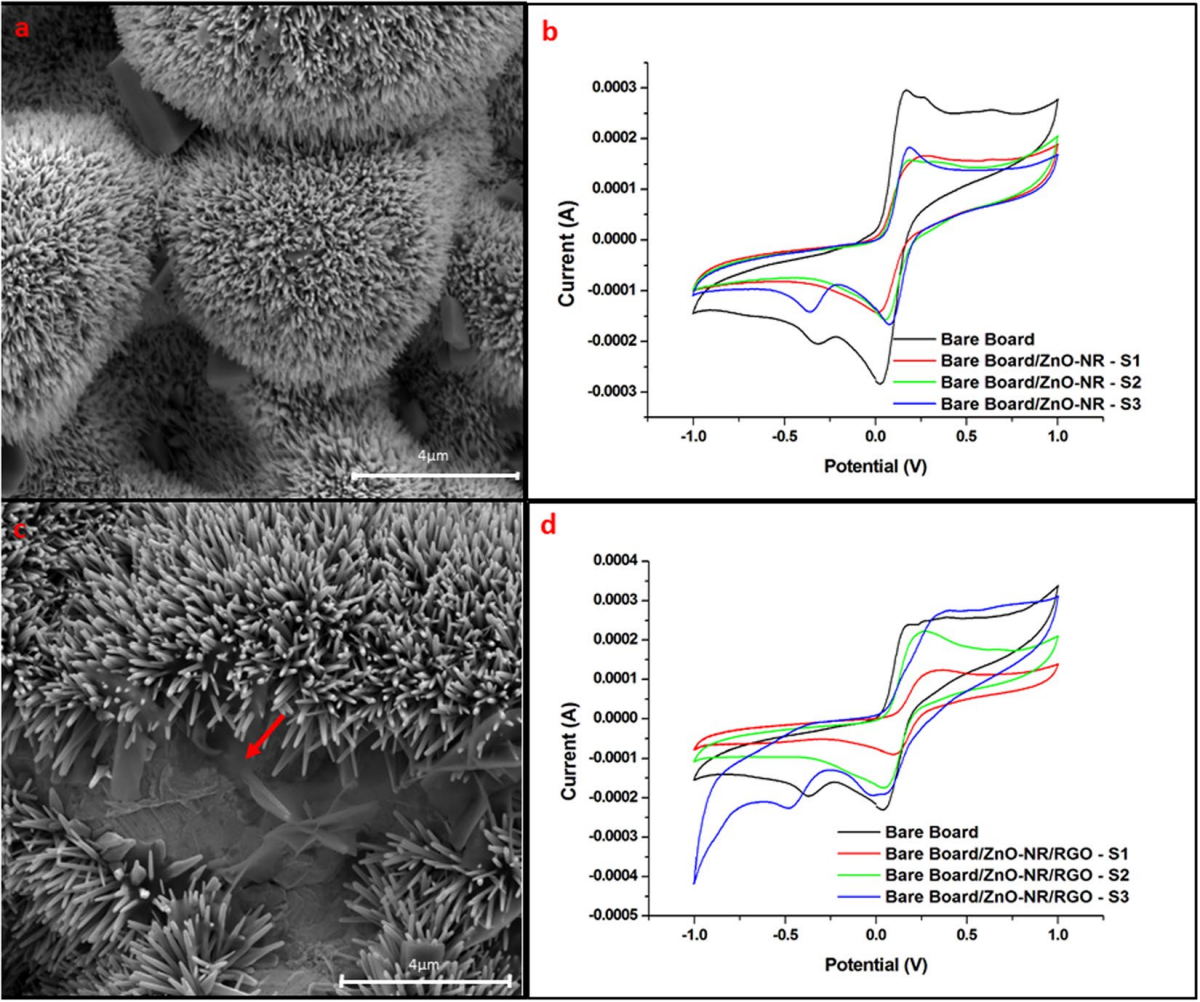
(**a**) SEM image of the ZnO NRs-based sensor board. (**b**) CVs of three different ZnO NR-based sensor boards (blue, red and green). (**c**) SEM image of the ZnO NRs/GO sheets composite-based sensor board. Red arrow indicates the GO sheets. (**d**) CVs of three different ZnO NRs/GO sheets composite-based sensor boards (blue, red and green). CVs in black in b and d are the calibration curves of the bare-sensor boards. All CVs were performed in the presence of K_3_[Fe(CN)_6_]/K_4_[Fe(CN)_6_] (10 mM) in NaNO_3_ (0.5 mol.L^−1^), with a scan rate of 100 mV.s^−1^.

The morphologies of the ZnO NRs and ZnO NRs:RGO composite were studied by SEM (Fig. 3a,c, respectively). The morphological difference between them is in the presence of the GO sheets on the surface of the ZnO NRs, indicated by the red arrows in Fig. 3c. Furthermore, a slight increase in the Ipa is observed for ZnO NRs:RGO compared to ZnO NRs (Fig. 3b,d, respectively). We believe that during the synthesis, the GO may be reduced to RGO. The RGO has superior conductivity, and its special concave-convex topography increases the number of electrochemical active sites, allowing the detection of weak signals^38^. Despite the higher Ipa for ZnO NRs:RGO, triplicate analyzes of the sensor boards show better reproducibility for ZnO NRs-based sensor boards. The coefficient of variation obtained for ZnO NRs-based sensor boards were 5.1% (Fig. 3d). Whereas, ZnO NRs:GO composite- based sensor boards showed a coefficient of variation of 25.2% of (Fig. 3b). The worst reproducibility of the composite-based sensor boards is due to the difficulty of controlling the same amount of GO/RGO sheets on the surface of the ZnO NRs. GO/RGO sheets tend to stack during the CBD. The control of simultaneous processes of non-staking and GO reduction is a challenge to be overcome. For this reason, we choose to work with the ZnO NRs-based sensor boards for making biosensors for detecting 8-OHdG.

### Anti-8-OHdG immobilization

Oriented immobilization of the antibodies on the sensor board is a crucial step in obtaining an immunosensor with high-performance and excellent specificity^28,39,40^. Modification of the ZnO NRs surface with Cys and Glut makes it, particularly susceptible to the immobilization of biomolecules. Here, all modifications on the surface of our ZnO NRs-based sensor board were accompanied by CV measurements (Fig. S2). As expected, we can observe a decrease of the Ipa as Cys and Glut are added on the ZnO NRs surface. The decrease in Ipa is related to insulating characteristics of the Cys, Glut, and Ab molecules.

**Figure 4:**
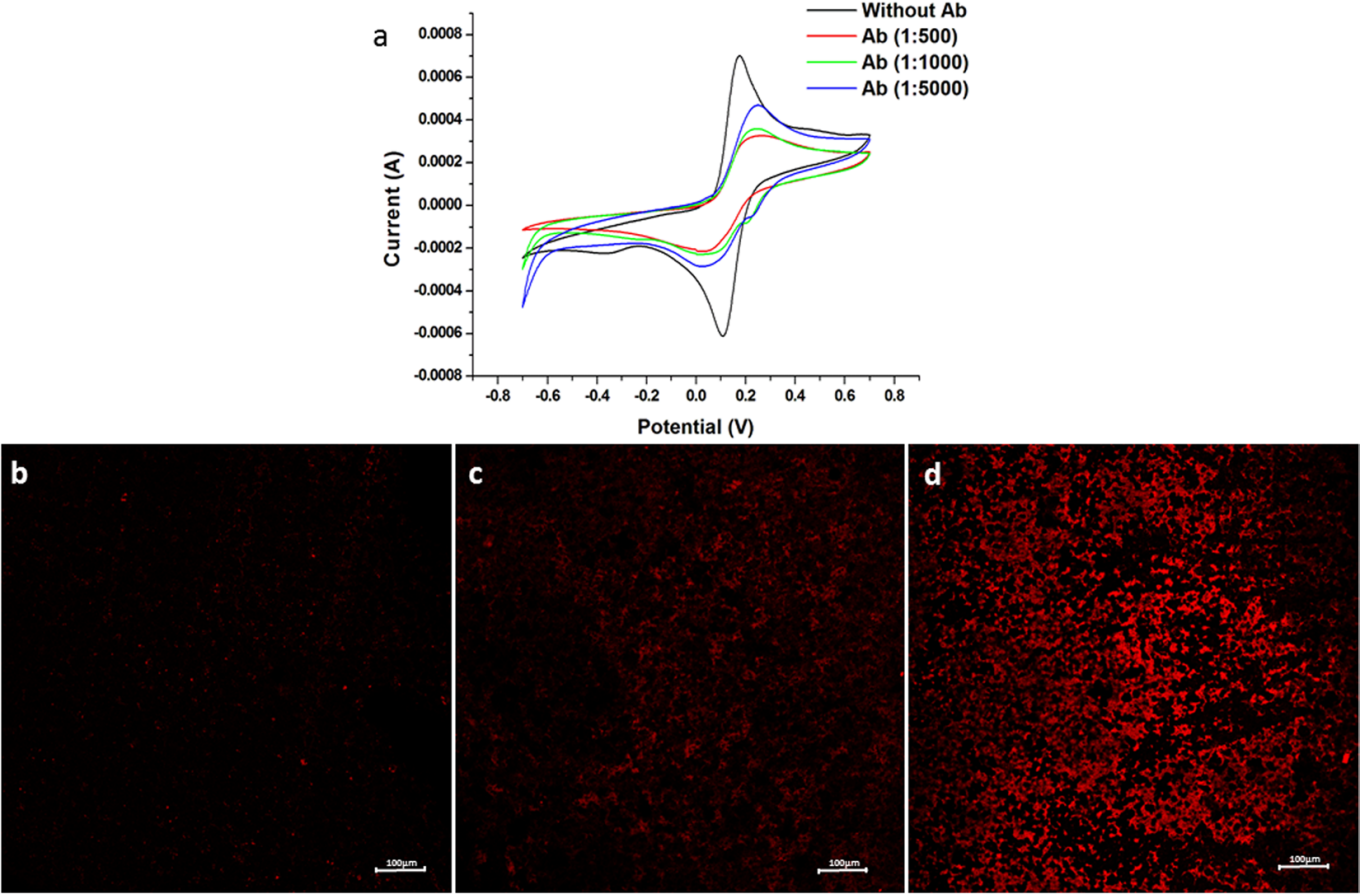
(**a**) CVs obtained for ZnO NRs-based sensor boards: black curve for sensor board without Ab immobilization. Red, green and blue curves for sensor board immobilized with Ab 1:500, 1:1000 and 1:5000, respectively. All CVs were performed in the presence of K_3_[Fe(CN)_6_]/K_4_[Fe(CN)_6_] (10 mM) in NaNO_3_ (0.5 mol.L^−1^), with a scan rate of 100 mV.s^−1^. (**b**–**d**) Fluorescence Confocal Micrographies. (**b**) Bare sensor board. (**c**) ZnO NRs-based sensor board. (**d**) ZnO NRs-based sensor board with antibody immobilization 1:5000. All sensors were incubated with fluorescent secondary antibody.

In order to avoid steric hindrance of antibody binding to ZnO NRs surface, as well as reduce the cost of the biosensor, we performed tests using three antibody concentrations (1:500; 1:1000 and 1:5000). As expected, a decrease of the anodic peak (Ipa) is observed with increasing antibody concentration (Fig. 4a). Nonetheless, Ipa values showed no significant difference at 1: 1000 and 1: 500 antibody concentrations (Fig. 4a). This indicates saturation of the active sites available for immobilization in concentration higher than 1:1000. When considering the Ipa value and the cost of antibody, we chose to use an anti-8OHdG concentration 1:5000 because it showed good sensitivity at a lower concentration.

Confocal Fluorescence Microscopy analyzes confirmed the immobilization of the antibody on the ZnO NRs-based sensor board (Fig. 4b–d). No fluorescence is observed for the bare sensor board (Fig. 4b). A small fluorescence is observed in the sensor board without Ab immobilization due to the ZnO reflecting part of the red spectrum (Fig. 4c). On the other hand, intense fluorescence is noted for a ZnO NRs-based sensor board with addition of the Ab (Fig. 4d) confirming its immobilization.

The stability of the immunosensor was assessed by ten successive CVs voltammograms performed in the presence of 10 mmol.L^−1^ of K_4_[Fe(CN)_6_] prepared in 0.5 mol.L^−1^ NaNO_3_ electrolyte at 100 mV.s-1 (Fig. S3a). The variation of redox peaks was low (9.7% of variation coefficient). The reproducibility of the immunosensor response was evaluating using four different sensors. Good reproducibility was obtained (coefficient of variation = 10.0%) (Fig. S3b). The low coefficient of variation for repeatability and reproducibility proves the excellent quality of our immunosensor.

### Immunosensor response to 8-OHdG

**Figure 5:**
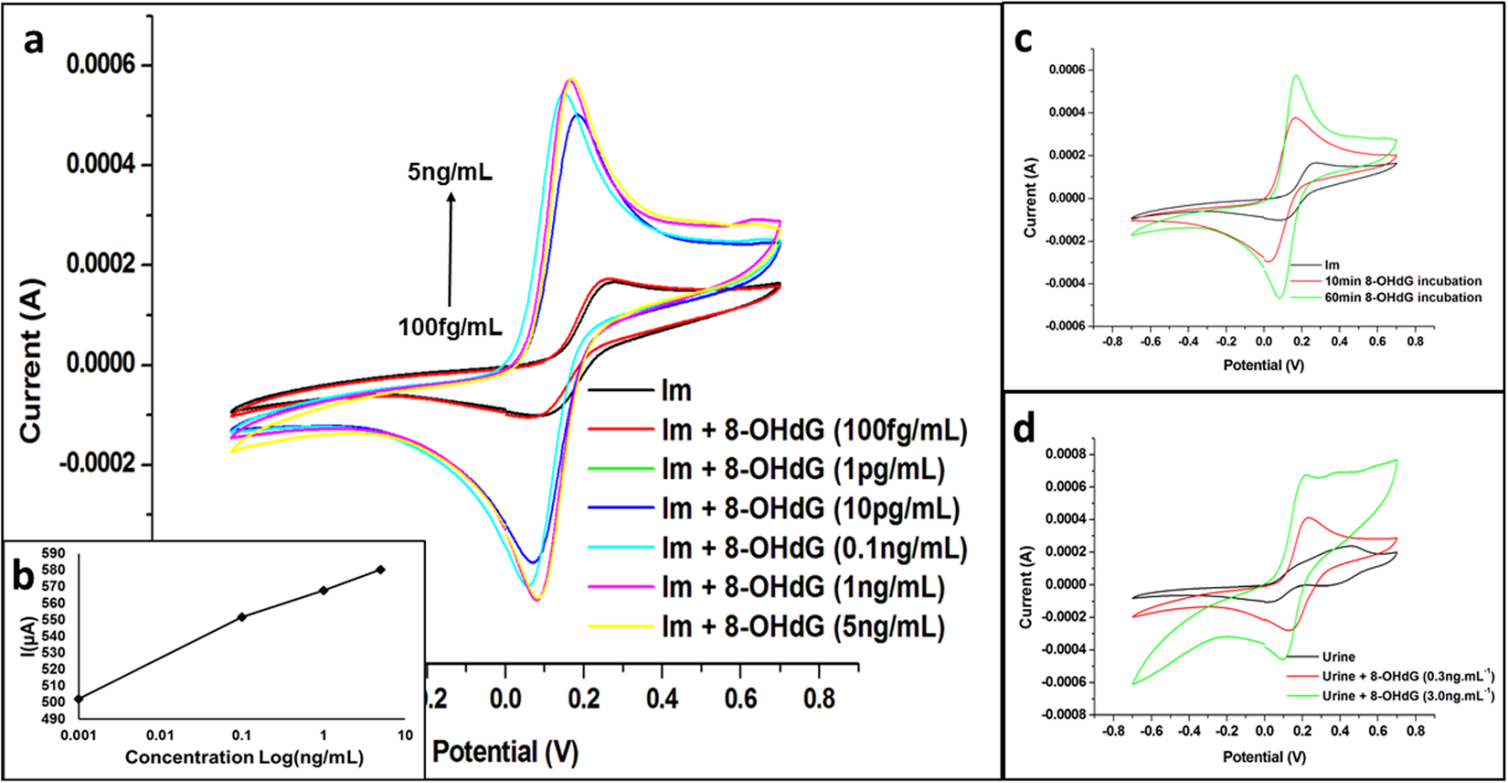
(**a**) CVs for immunosensors (Im) with different 8-OHdG concentrations. (**b**) Linearity of calibration curve obtained from the anodic peaks of three replicated measurements. R^2^ = 0.9898; n = 4. (**c**) 8-OHdG immunosensor (Im) incubated with 8-OHdG standard (5 ng.mL^−1^) at different times: in black without incubation; in red and green 10 min and 60 min of 8-OHdG incubation. (**e**) 8-OHdG imunosensor (Im) incubated with urine samples. CV in red is just urine standard; in green and blue urine with addition of 0.3 ng/mL 8-OHdG and 3.0 ng/mL, respectively. All CVs were performed in the presence of K_3_[Fe(CN)_6_]/K_4_[Fe(CN)_6_] (10 mM) in NaNO_3_ (0.5 mol.L^−1^), with a scan rate of 100 mV.s^−1^.

Under optimized experimental conditions, the calibration curve of immunosensor was obtained. The electrodes were incubated at different concentrations of 8-OHdG and subjected to CV measurement in the presence of K_3_[Fe(CN)_6_]/K_4_[Fe(CN) _6_] (10 mM) in NaNO_3_ (0.5 mol.L^−1^). The results show that the anodic peak increases as 8-OHdG antigen concentration does (Fig. 5a). This means that binding of 8-OHdG antigen with the antibody occurs through release of the electrons. In sensing mechanism, the oxygen species present in the surface of the ZnO NRs binds to cys and provides amino groups on the surface of them. These groups bind to glut aiming to get carbonyl groups, which are easily bound to amino groups present in the anti- 8-OhdG molecule. Finally, the antigen binding site present in the antibody molecule allows binding to the 8-OhdG antigen. After binding, 8-OHdG antigen releases electrons assisting to reduce [Fe(CN)_6_]^3−^ into [Fe(CN)_6_]^4−^. The [Fe(CN)_6_]^4−^ ions are again oxidized to [Fe(CN)_6_]^3−^ during forward CV scan, and the transfer of electrons occur via ZnO NRs.

Linearity in the calibration curve was obtained over the range 1 pg.mL^−1^ to 5 ng.mL^−1^ of 8-OHdG (R^2^ = 0.9898; n = 4) (Fig. 5b). The immunosensor developed during this study showed a Limit of Detection of 100 fg.mL^−1^ (Fig. S4).

Commercial ELISA Kits for 8-OHdG show a limit of detection equal to 0.1 ng.mL^−1^. The immunosensor developed during this study showed as lower concentration detected in linear range of 1 pg.mL^−1^ (Fig. 5a). This limit is one hundred times less than ELISA test and better than other reported biosensors (Table S1)^22–24,26,27,41–45^. Our immunosensor shows linear range 3 pM to 17.6 nM and the limit of detection was found to be 0.3 pM (100 fg/mL). Besides, previous studies^10,46^ showed the concentration of 8-OHdG in serum samples is 0.2 ng.mL^−1^ for health patients, 0.6 ng.mL^−1^ for pre-diabetics, and 2 ng.mL^−1^ for diabetics. In this way, our immunosensor is capable of detecting 8-OHdG in urine within a full range: healthy or with physiological change. The better properties of our immunosensor show the importance of controlling its parameters and characteristics during manufacturing process.

In order to find the optimum incubation period of 8-OHdG, different times (10 and 60 minutes) were tested. A higher Ipa is obtained in 60 min of incubation (Fig. 5c) meaning better charge transfer process during linkage of the antigen. Accordingly, one hour could be more efficient for improving the performance of the immunosensor.

Urine samples were assayed, and recovery experiments were carried out via the standard addition method. For representing the 8-OHdG concentration found in health and diabetic patients, the urine samples were spiking with 0.3 and 3.0 ng.mL^−1^. Assuming a null concentration of 8-OHdG in the urine sample, the CVs show a recovery of 8-OHdG molecules spiked in the urine (Fig. 5d). Clearly, higher I_pa_ values are obtained as 8-OHdG concentration increases. However, CVs show slight differences for the immunosensor incubated with human urine and without it. These differences may be related to presence of other compounds in the urine, as well as; 8-OHdG produced physiologically^6^. If we compare with the immunosensor, an increase of Ipa of 42.76% in the presence of the compounds contained in the urine is observed. However, this increase is 151.77% with addition of 0.3 ng.mL^−1^ and 278.95% for 3 ng.mL^−1^. It is important to remember that humans physiologically produce 8-OHdG, not only pathogenesis^8^. This means that the small increase observed in presence just of urine, without addition of 8-OhdG, should be correlated with its physiological presence in it.

These results prove the electrochemical immunosensor developed here show lower limit of detection, higher specificity and selectivity, and excellent reproducibility. Another advantage of our immunosensor is the possibility to detect 8-OHdG by using no invasive analysis, just by collecting the urine.

## Conclusions

In the present work, a sensor board was developed using a simple PCB technology. Stable and reproducible sensor boards are made by controlling the thickness of the gold working electrode. For aiding posteriori immobilization of the antibody on WE, ZnO NRs or ZnO NRs:RGO composites were grown on it. ZnO NRs-based sensor boards showed better reproducibility than ZnO NRs:RGO composite-based sensors boards.

The anti-8-OHdG antibody was successfully immobilized on the ZnO NRs surface. The incubation period and the amount of the antibody were controlled to ensure a better charge transference process, and reduced the cost of electrochemical immunosensors. Our immunosensor showed sensitivity and selectivity to 8-OHdG with detection capacity in the range of 0.001–5.00 ng.mL^−1^. Our immunosensor shows the great advantage for testing biological samples, such as urine, without any previous sample preparation. It can respond electrically to the chemical stimulus with sensitivity, selectivity, reproducibility and low cost. It could act as predictive biomarker of oxidative stress involved in illness, such as: cancer, diabetes and neurodegenerative diseases.

## Materials and Methods

### Electrochemical bare-sensor board fabrication

The bare-sensor board was made on FR-4 (1.6 mm thick) sheets using a Printed Circuit Board Technology (PCB). Two different metals, Copper (Cu) and gold (Au) films, were used as working and counter electrodes. The electrodes were configured each to be electrically isolated from the others. For copper electrodes, we used a 1/1 OZ FR-4 copper-clad sheet available commercially (Jiangsu Sunyuan Aerospace Material Co., LTDA). For gold electrodes, we deposited gold films with different thickness on FR-4 sheets by an electrolytic method. The reference electrode was deposited by screen printed by using Silver Conductive Epoxy, H2OE EPO-TEK. After that, it was cured at 100 °C for 2 h.

### Preparation of seeding layer

Zinc acetate (ZnAc) and graphene oxide (GO) seed layers were prepared using a 30 mmol.L^−1^ ZnAc ethanolic solution, 0.05 g.L^−1^ GO sheets solution. The seed layers were deposited by Spray Coating (Exacta Coat), using 5 W, 1.00 KPa and 0.30 mL.min^−1^ at 100 °C. Three different kinds of samples were prepared by changing the number of layers deposited by spray. The sample named 03GO03ZnAc consists of spraying 3 layers of a GO solution followed by 03 layers of ZnAc solution. 06GO06ZnAc is composed of 06 sprayed layers of a GO solution followed by 06 layers of ZnAc solution; and 12GO12ZnAc refers to spray 12 layers of GO solution followed by 12 layers of ZnAc solution. After deposition of seeding layers, samples were used for growing ZnO NRs and ZnO NRs: GO composite by CBD.

### Growth of ZnO NRs and ZnO NRs:RGO composite by chemical bath deposition

The samples were synthesized by CBD as previously described by Vessalli^32^. Briefly, we added hexamethylenetetramine (HMTA, Sigma-Aldrich) and zinc nitrate (Zn(NO_3_)_2_, Sigma-Aldrich) in the proportion 1:1 in a Polytetrafluoroethylene (PTFE) vessel. For composite synthesis, a 0.0125 g.L-1 GO solution was also added to precursor solution. After that, the bare-sensor board was immersed in the solution. The PTFE vessel was placed in silicone bath. The solution was stirred and heated at 90 °C for 2 h, aiming to promote the growth of ZnO NRs and ZnO NRs:RGO composite.

### Immobilization of the anti-8-OHdG

**Figure 6:**
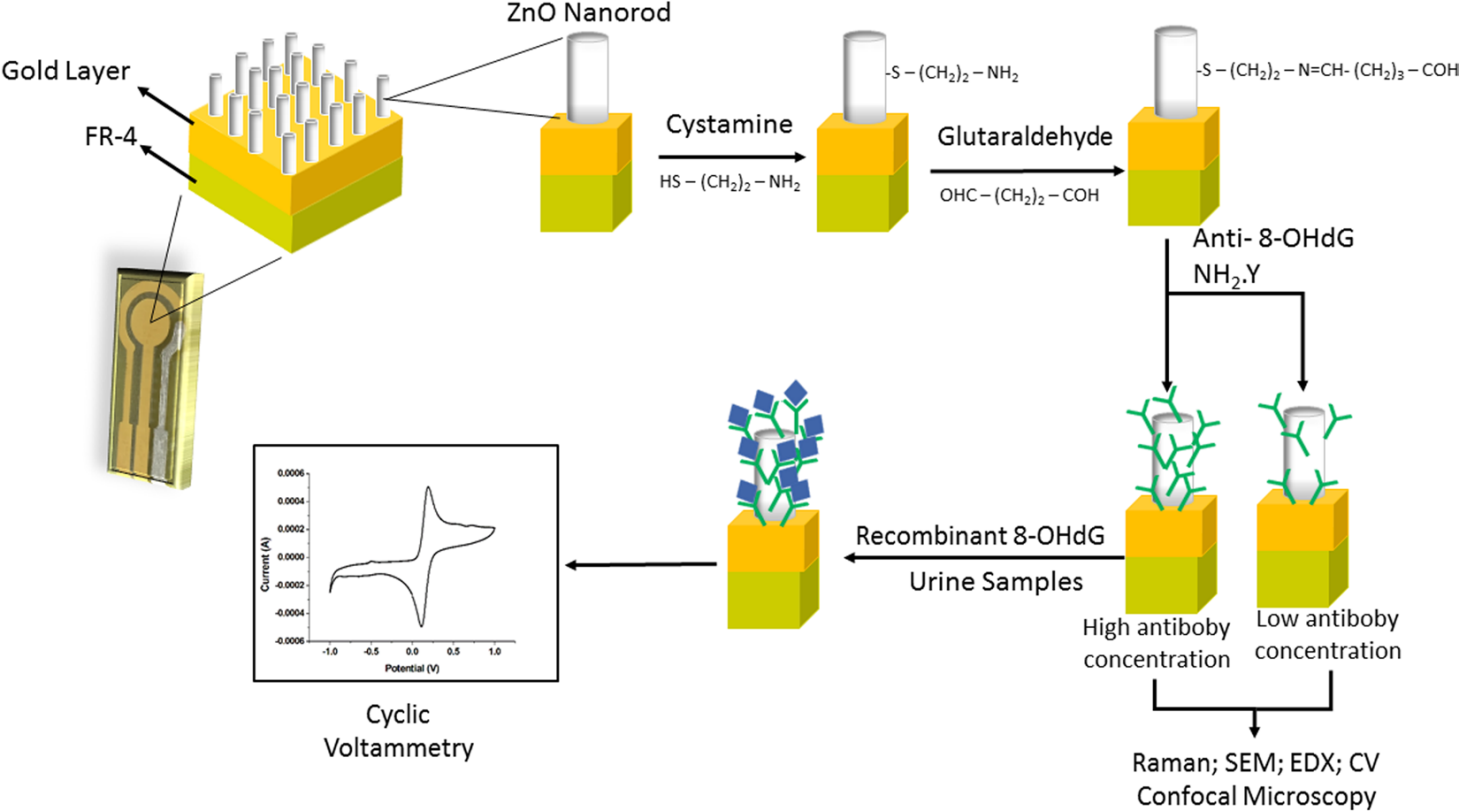
Schematic illustration of the stepwise preparation of the 8-OHdG immunosensor.

The anti-8-OHdG antibody (Abcam (Cambridge, MA, USA)) was immobilized via 20 mM Cystamine dihydrochloride (Cys) (Alfa Aesar) and 2.5% Glutaraldehyde (Glut) (Electron Microscopy Sciences) on the surface of ZnO nanorods (ZnO-NRs). Firstly, Cys binds to oxygen dominant species on ZnO NRs surface, and modified it with amino groups. Subsequently, Glut binds to Cys and providing carbonyl group on the surface of the sensor, so that the amino group of the antibody (NH2-Y) is readily bound. Twenty microliters of anti-8-OHdG monoclonal antibody solution were diluted in 0.1 M buffered phosphate saline (PBS) pH 7.4. Three different concentration of the solution (1:5000, 1:1000 and 1:500) was dropped on the surface of the working electrode and incubated for 12 hours at 4 °C. The incubation experiment was performed in a moist chamber. After that, the samples were evaluated by Cyclic Voltammetry (CV) analyzes. Figure 6 shows an overview of the stepwise preparation of the immunosensor.

### Electrochemical assays

The analytical responses of the immunosensor were evaluated by electrochemical measurements by using cyclic voltammetry (CV). During CV assays, potential was scanned from −0.7 to 0.7 V at the scan rate of 100 mV.s^−1^ recorded in a solution of 10 mmol.L^−1^ K_4_[Fe(CN)_6_] and 0.5 mol.L^−1^ NaNO_3_ as a mediator. All experiments were conducted in triplicate at room temperature.

### Characterization methods

All samples were characterized by Scanning Electron Microscopy (SEM) in an Inspect F50 SEM microscope and Raman spectroscopy in a Confocal Raman model Horiba T64000 spectrometer. For Raman analyzes, we used an exposure time of 30 s, accumulation of 10 spectrums and LASER source of 633 nm. Immunofluorescence Confocal Microscopy was used to identify the antibody binding to the sensor after immobilization. For the Immunofluorescence Confocal Microscopy analysis, the sensors were incubated during 1 h with Alexa Fluor 594 (Life Technologies) fluorescent secondary antibody using 1:500 ratio, and room temperature. The sensors were then evaluated by fluorescence intensity in a confocal microscope Leica, model TCS SP5 II. The experiments were performed in a moist chamber.

### Immunosensor performance for 8-OHdG detection

After immobilization, the immunosensor was evaluated by its calibration curve and limit of detection (LoD). The 8-OHdG standard (Cayman Chemical) was incubated to perform all analyzes according to protocol described below. First, immobilization of the anti-8-OHdG antibody occurs on ZnO NRs surface followed by washing of the sensor boards with PBS. Second, different concentrations of 8-OHdG antigen (from 0.0001 to 5.0 ng/m L^−1^) are incubated at room temperature followed again for washing with PBS buffer. The time of 8-OHdG antigen incubation was analyzed by incubation 8-OHdG (5.0 ng/m L^−1^) for 10 and 60 min. The experiments were performed in a moist chamber. The evaluation was performed by CV analysis.

### Analyzes in urine samples

The urine samples used here were supplied by the author herself. All methods were carried out in accordance with guidelines and regulations of the National Committee on Research Ethics, CONEP/CNS/MS, of the Brazil. All experimental protocols were approved by Comissão Nacional de Ética em Pesquisa – CONEP/CNS/MS. Selectivity of the 8-OHdG immunosensor was assessed promptly in human urine samples. The human urine samples were collected in sterile and immediately frozen at −20 °C. Before analysis, the samples were diluted in PBS buffer, in a 1:10 ratio, following by the spike of 8-0HdG with 02 established concentrations (0.3 ng/mL^−1^ and 3.0 ng/mL^−1^). The sensors were incubated in the samples during 60 min and characterized by CV analysis.

## Supplementary information

Supplementary Information
