# Author Correction: Controlling parameters and characteristics of electrochemical biosensors for enhanced detection of 8-hydroxy-2′-deoxyguanosine

**DOI:** 10.1038/s41598-020-60495-4

**Published:** 2020-02-27

**Authors:** Aline M. Faria, Elisa B. M. I. Peixoto, Cristina B. Adamo, Alexander Flacker, Elson Longo, Talita Mazon

**Affiliations:** 1 Centro de Tecnologia da Informação Renato Archer, CTI, Rod. D. Pedro I, KM 143.6, 13069-901 Campinas, SP Brazil; 2 CDMF, Universidade Federal de São Carlos, P.O. Box 676, São Carlos, SP 13565-905 Brazil

Correction to: *Scientific Reports* 10.1038/s41598-019-43680-y, published online 15 May 2019

The original version of this Article contained errors in the spelling of the authors Cristina B. Adamo and Alexander Flacker, which were incorrectly given as Cristiane B. Adamo and Alexandre Flacker respectively. These errors have now been corrected in the PDF and HTML versions of the Article, and in the accompanying Supplementary Information file.

