# Supplementary material for: Controlling parameters and characteristics of electrochemical biosensors for enhanced detection of 8-hydroxy-2′-deoxyguanosine
Source: Sci Rep. 2019 May 15;9:7411. doi: 10.1038/s41598-019-43680-y (PMC6520373; doi:10.1038/s41598-019-43680-y)
Supplement: Supplementary file 1 — Supplementary Information [file 41598_2019_43680_MOESM1_ESM.doc]

***Supplementary Information***

***Controlling parameters and characteristics of electrochemical biosensors for enhanced detection of 8-hydroxy-2'-deoxyguanosine***

Aline M. Faria^1^, Elisa B.M.I Peixoto^1^, Cristiane B. Adamo^1^, Alexandre Flacker^1^, Elson Longo^2^, Talita Mazon^1^*

Centro de Tecnologia da Informação Renato Archer, CTI, Rod. D. Pedro I, KM 143.6, 13069-901, Campinas, SP, Brazil

CDMF, Universidade Federal de São Carlos, P.O. Box 676, São Carlos, SP, 13565-905, Brazil

* Correspondence and requests for materials should be addressed to T.M (email: [talita.mazon@cti.gov.br](mailto:talita.mazon@cti.gov.br))


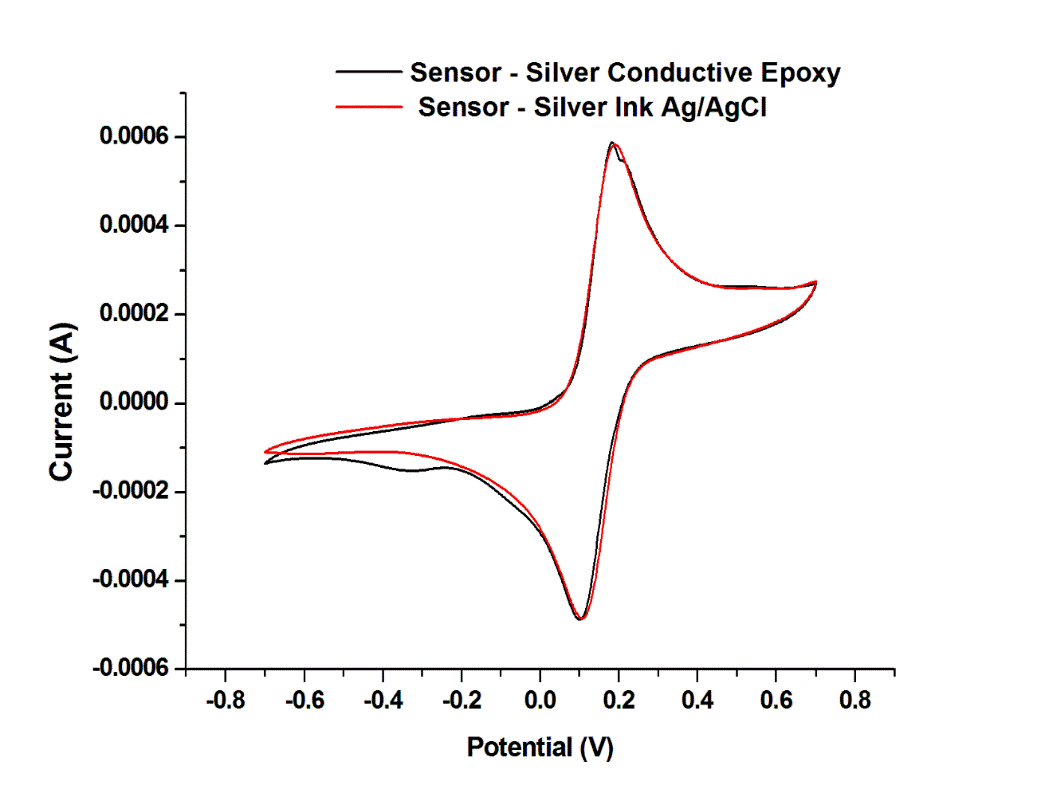


**Figure S1.** Cyclic voltammograms (CVs) obtained of sensor using silver conductive epoxy (black) in reference electrode and sensor using ink Ag/AgCl (red) in reference electrode. All CVs were performed in the presence of K_3_[Fe(CN)_6_]/ K_4_[Fe(CN)_6_] (10mM) in NaNO_3_ (0.5 mol.L^-1^), with a scan rate of 100mV.s^-1^.


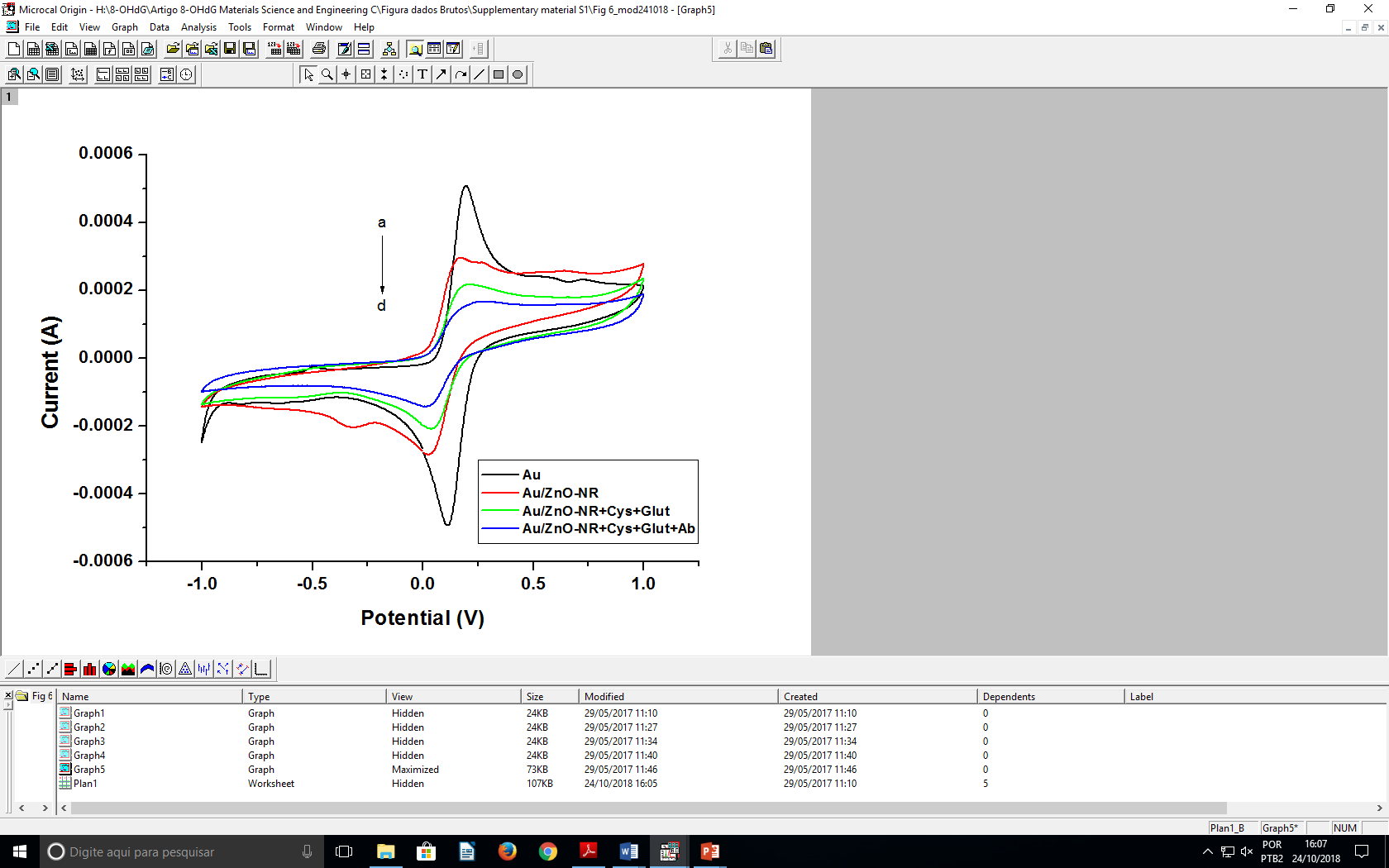


**Figure S2.** CVs obtained after each modification on the surface of bare sensor board: a) in black for bare sensor board made by using Au film-based WE. b) in red for ZnO NRs grew on Au film-based WE (ZnO NRs-based sensor board). c) in green for ZnO NRs-based sensor board modified with cystamine (Cys) and glutaraldehyde (Glut). d) in blue for ZnO NRs-based sensor board modified with cystamine (Cys), glutaraldehyde (Glut), and antibody (Ab). All Cvs were performed in the presence of K_3_[Fe(CN)_6_]/ K_4_[Fe(CN)_6_] (10mM) in NaNO_3_ (0.5 mol.L^-1^), with a scan rate of 100mV.s^-1^.


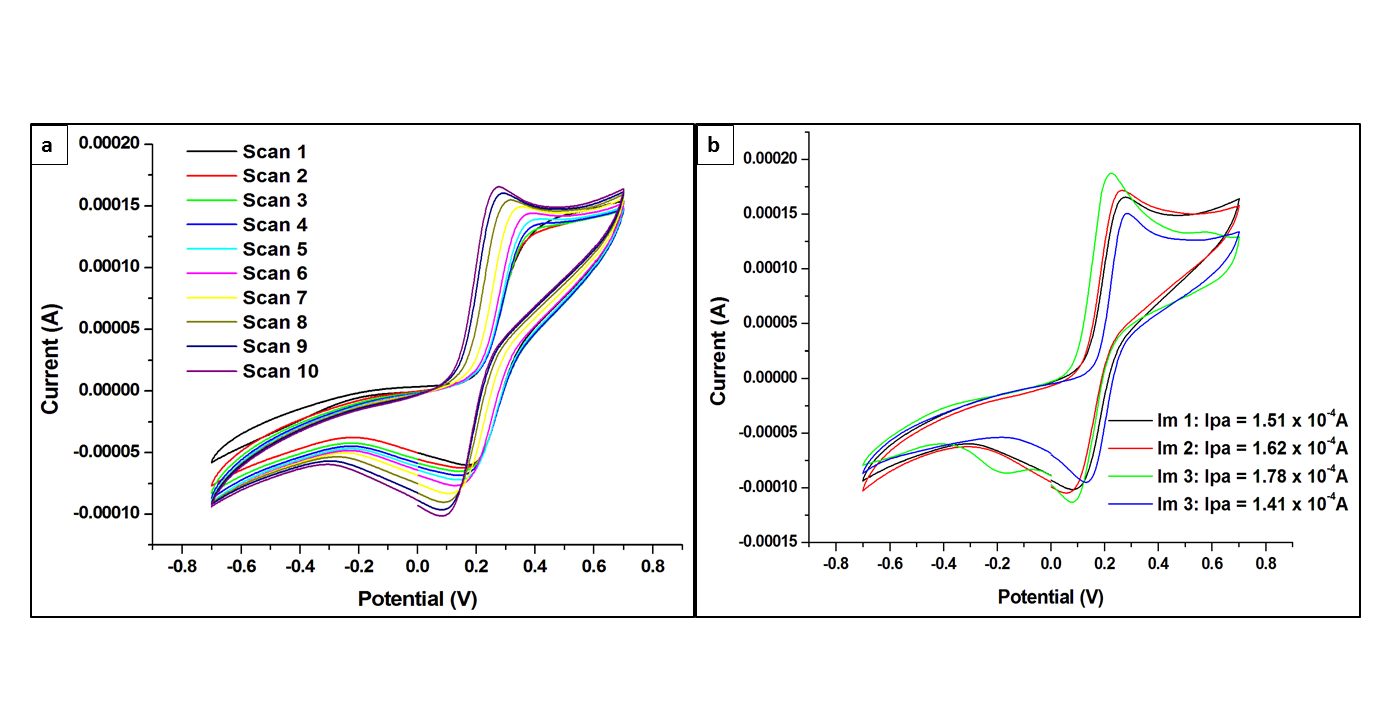
**Figure S3.** a) Immunosensor with 10 curves. b) CV of Four different immunosensors (Im). All CVs were performed in the presence of K_3_[Fe(CN)_6_]/ K_4_[Fe(CN)_6_] (10mM) in NaNO_3_ (0.5 mol.L^-1^), with a scan rate of 100mV.s^-1^.


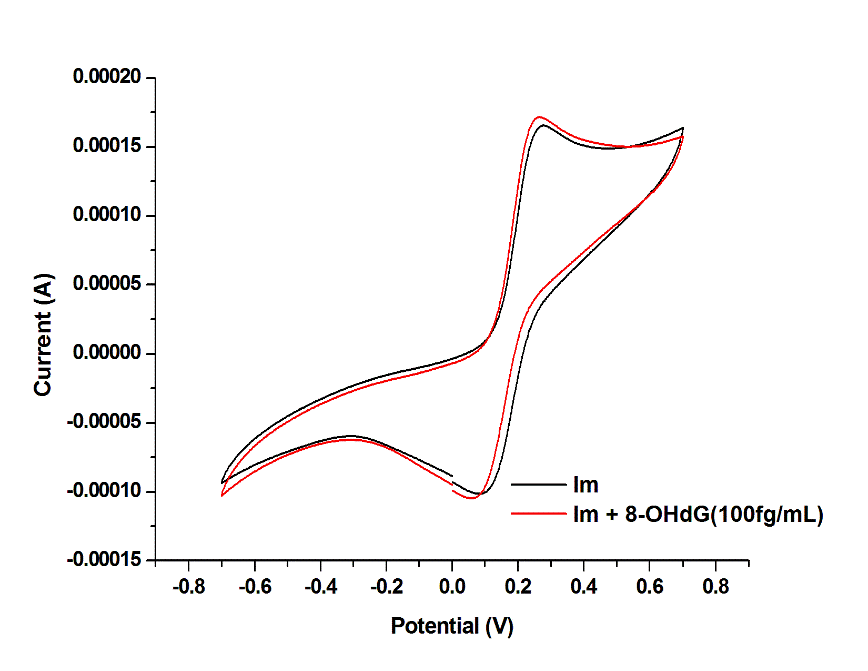


**Figure S4.** CVs for immunosensor (Im) (black) and red immunosensor incubated with 8-OHdG (100fg/mL). All CVs were performed in the presence of K_3_[Fe(CN)_6_]/ K_4_[Fe(CN)_6_] (10mM) in NaNO_3_ (0.5 mol.L^-1^), with a scan rate of 100mV.s^-1^.

**Table S1.** Electrochemical biosensors to detect 8-OhdG reported in the literature.

| **Article** | **Detection** | **Working eletrode** | **Linear range** | **Analysis of biologic Samples** |
| --- | --- | --- | --- | --- |
| Gutiérrez, A. et al 2013 ^23^ | Electrochemical | carbon nanotubes (SWCNT) functionalized  with L-lysine / glassy carbon electrodes (GCE) | 3.0 x 10^-7^ M and  1.0 x 10^-5^ M | No |
| Jia, L. and Wang, H., 2013^24^ | Electrochemical | Graphene/Nafion Nanocomposite Film | 0.07 - 33.04 μM | No |
| Tehrani, Z. et al 2014^27^ | Electrochemical | Micro-channels  of chemically modified multi-layer epitaxial graphene (MLEG) | concentrations as low as 0.1 ng ml−1  (0.35 nM) | No |
| Ferreira, N.S.; Sales, M.G.F, 2014 ^42^ | Electrochemical | Gold modified platform | 0.02 - 7.0 ng/mL | Yes urine |
| Yang L, et al 2015^22^ | Electrochemical | SWCNTs-Nafion film | 0.03 to 1.25 𝜇M | Yes urine |
| Jia, L.P. et al , 2015 ^26^ | Electrochemical | single-stranded DNA (ss-DNA)/  graphene nanosheets(GNs) | 0.0056–1.155 μM, 1.155–11.655 μM and11.655–  36.155 μM, | Yes urine |
| Pan, D. et al , 2015^43^ | Electrochemical | Glassy carbon electrode  modified with chitosan and poly(indole-5-carboxylic acid | 0.1 to 10,000 ng·mL^−1^ | Yes urine |
| Guo et al, 2016^44^ | Electrochemical | multi-walled carbon  nanotubes (MWCNTs) modified glassy carbon electrode | 5.6x10^-8^ to 6.1x10^-6^ M and  6.1x10^-6^ to 1.6x10^-5^ M, | Yes urine |
| Martins, G.V . et al, 2017^45^ | Electrochemical | nanostructured carbon + polymer PEDOT | 50–1000 ng/ml | Yes serum |
| Khan, M.Z.H. et al, 2018^46^ | Electrochemical | poly (L-arginine) /graphene wrapped Au nanoparticles /  glassy carbon electrode | 1. to 100 nM | Yes urine |
